# Polycystins and Mechanotransduction in Human Disease

**DOI:** 10.3390/ijms20092182

**Published:** 2019-05-02

**Authors:** Antonios N. Gargalionis, Efthimia K. Basdra, Athanasios G. Papavassiliou

**Affiliations:** Department of Biological Chemistry, Medical School, National and Kapodistrian University of Athens, 11527 Athens, Greece; agargalionis@yahoo.gr (A.N.G.); ebasdra@med.uoa.gr (E.K.B.)

**Keywords:** mechanotransduction, polycystin, cancer, metastasis, cyst formation, osteoblast differentiation, psoriasis, cardiomyopathy

## Abstract

Alterations in the process of mechanotransduction have been implicated in the pathogenesis of several diseases such as genetic diseases, osteoporosis, cardiovascular anomalies, and cancer. Several studies over the past twenty years have demonstrated that polycystins (polycystin-1, PC1; and polycystin-2, PC2) respond to changes of extracellular mechanical cues, and mediate pathogenic mechanotransduction and cyst formation in kidney cells. However, recent reports reveal the emergence of polycystins as key proteins that facilitate the transduction of mechano-induced signals in various clinical entities besides polycystic kidney disease, such as cancer, cardiovascular defects, bone loss, and deformations, as well as inflammatory processes like psoriasis. Herewith, we discuss data from recent studies that establish this role with potential clinical utility.

## 1. Introduction

Cells respond to a series of mechanical signals, and experience mechanical stresses from the extracellular and intracellular environments that regulate normal cell function, stem cell differentiation, and tissue homeostasis [1]. Mechanical stresses are formed from extracellular applied forces, cell-generated forces in the cytoskeleton, and alterations in substrate mechanics. Therefore, a cell can be subjected to various types of forces according to the tissue it belongs to. Such forces are the external tensile forces which are formed from alterations of the extracellular matrix (ECM) stiffness, compressive forces, contractile forces that develop internally by the cytoskeleton, hydrostatic pressure, and forces in the form of shear stress due to fluid flow over the cell [2]. The process by which the cells convert the resultants of these forces into biochemical signals in order to elicit their response is termed “mechanotransduction”. Mechanotransduction is mediated by its own mechanotransducing machinery, a broad mechanical network that includes structures such as stretch-activated ion channels, the proteins of the glycocalix in epithelial cells, the proteins of the cell-to-cell adhesion and cell-to-ECM focal adhesion complexes, the unfolded ECM proteins, cytoskeletal elements (filaments, crosslinkers, motor proteins), G protein-coupled receptors (GPCR), etc. [3,4].

We now know that in a series of pathophysiologies such as atherosclerosis, cancer, osteoporosis, and deafness, as well as in several developmental disorders, the aberrant responses to applied or cell-generated mechanical cues contributes to the development and progression of the diseases [3]. This group of diseases shares common features of altered mechanotransducing signaling, and therefore it is crucial to identify the components of such complexes that mediate this process. In this context, polycystins are mechanosensing proteins that have been long ago attributed the role of being the causative agent of the autosomal polycystic kidney disease (ADPKD), where they have been extensively investigated. However, recent studies reveal the participation of these proteins in several clinical entities which have been associated with distorted mechanotransduction (Table 1). In this review, we critically discuss polycystin-mediated molecular mechanisms in certain diseases, based on recent data.

## 2. Structure and Function of Polycystins

**Figure 1:**
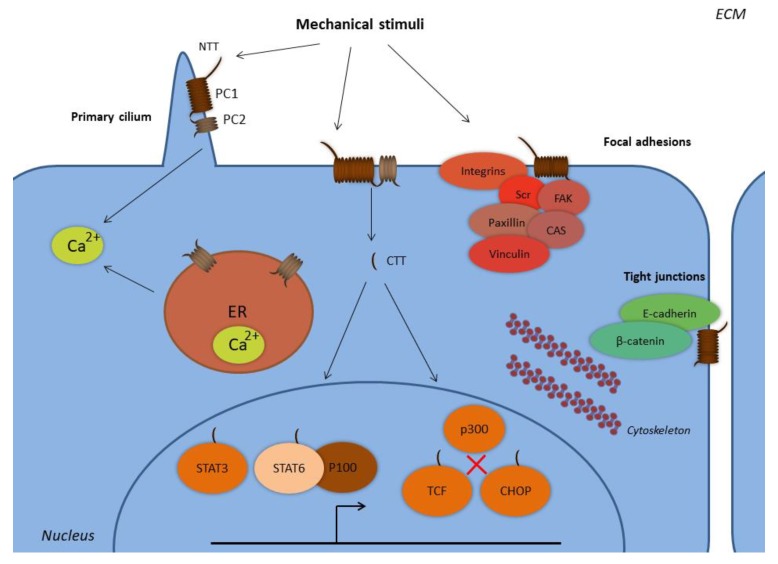
Subcellular localization of polycystins. Abbreviations: CHOP, C/EBP homologous protein; CTT, C-terminal tail; ER, endoplasmic reticulum; FAK, focal adhesion kinase; NTT, N-terminal tail; PC1, polycystin-1; PC2, polycystin-2; STAT, signal transducer and activator of transcription; TCF, T-cell factor. PC1 and PC2 form complexes at the primary cilium and at the plasma membrane, where PC1 senses extracellular mechanical stimuli with its N-terminal tail. PC1 is expressed at the focal adhesion complexes regulating cell-to-ECM interactions, along with proteins such as integrins, Src, focal adhesion kinase (FAK), paxillin, p130Cas, and vinculin. PC1 is also expressed in complexes with E-cadherin and β-catenin at the tight junctions regulating cell-to-cell interactions. The PC1 C-terminal tail is subjected to proteolytic cleavages and translocates to the nucleus, where it forms complexes with the transcription factors STAT3 and STAT6, and the coactivator p100. The PC1 CTT interacts with TCF and CHOP transcription factors in order to abolish their interactions with the p300 coactivator. PC2 is also expressed at the endoplasmic reticulum and regulates the concentration of the intracellular calcium.

Polycystins represent a family of proteins that belongs to the mechanotransducing machinery of the mechanotransduction process. Polycystins are expressed in a variety of epithelial cells, and the main representatives of the family are polycystin-1 (PC1) and polycystin-2 (PC2). PC1 is a 450 kDa transmembrane protein that functions as non-typical GPCR. PC1 consists of a large extracellular N-terminal end, 11 transmembrane domains, and an intracellular C-terminal end. Apart from the plasma membrane, PC1 is also expressed in epithelial protrusions called primary cilia (Figure 1). PC2 is a smaller protein (110 kDa) that functions as an ion channel permeable for calcium ions (Figure 1). PC2 is detected on the endoplasmic reticulum and the plasma membrane (Figure 1) [5,6]. The two proteins form heterotetrameric complexes with a 1:3 PC1:PC2 stoichiometry [7,8].

Polycystins exert several cellular functions through which they mediate mechanotransduction. PC1, either alone or with PC2, is located in cell-to-cell and cell-to-ECM protein complexes, and therefore it regulates the interactions among adjacent cells, and between cells and their surrounding ECM (Figure 1) [9,10,11,12]. However, PC1 does not only transduce extracellular physical cues into the cell interior as a receptor, but also applies direct transcriptional regulation through its C-terminal end. PC1 is subjected to several proteolytic cleavages, with at least three C-terminal tail (CTT) cleavages. The generated fragments translocate to the nucleus and function as transcriptional regulators (Figure 1) [13]. One CTT cleavage fragment interacts with the transcription factors signal transducer and activator of transcription 3 (STAT3), STAT6, and the co-activator p100 [14]. There is also a *γ*-secretase-mediated cleavage of PC1 that influences the activity of the T-cell factor (TCF) and C/EBP homologous protein (CHOP) transcription factors. The CTT of PC1 can interact with TCF and CHOP and prevent interactions between these transcription factors and the transcriptional co-activator p300 [13,15]. Recent studies also document that polycystins regulate mitochondrial function. In fact, polycystins sense oxygen levels, which in turn determine the subcellular topology and activity of the polycystin complex through its interaction with the O_2_-sensing prolyl hydroxylase domain-containing protein EGLN3 (PHD3). Cells with downregulated PC1 expression use less oxygen and exhibit lower levels of mitochondrial Ca^2+^ following bradykinin-induced endoplasmic reticulum Ca^2+^ release. This mechanism indicates that PC1 can modulate mitochondrial activity [16]. A recent study also showed that *Polycystic Kidney Disease 1* (*PKD1*)^−/−^ knockout renal epithelial cells depict aberrant fatty acid utilization, impaired morphology and function of mitochondria, and that mitochondria in kidneys of ADPKD patients have morphological alterations. These are caused by a PC1 CTT cleavage fragment that translocates to the mitochondria matrix [17].

## 3. Polycystins Govern Cyst Formation

ADPKD is the most common inherited disease of the kidney and it is characterized by mutations either on the *PKD1* gene (80–85%) or on the *PKD2* gene (15–20%) (Table 1). Mutations on the polycystin-encoding genes cause the overwhelming majority of loss-of-function mutations. Truncating *PKD1* mutations exhibit the poorest kidney survival, followed by non-truncating *PKD1* and *PKD2* mutations, respectively [18]. Initially, the “two-hit” hypothesis was the proposed mechanism for the development of ADPKD, which suggested that a complete loss of the normal allele has to occur for the onset of the disease [19,20]. However, the complexity of the cyst formation and respective studies highlighted the threshold model for cystogenesis in ADPKD, according to which cysts are formed when the functions of polycystins fall below a specific dosage [21,22]. Apart from loss-of-function cases of polycystic kidney disease, there are also studies which demonstrate the formation of cysts in transgenic mice following *PKD1* overexpression, and also kidney tubular aberrations after *PKD2* overexpression in mice [23,24].

**Figure 2:**
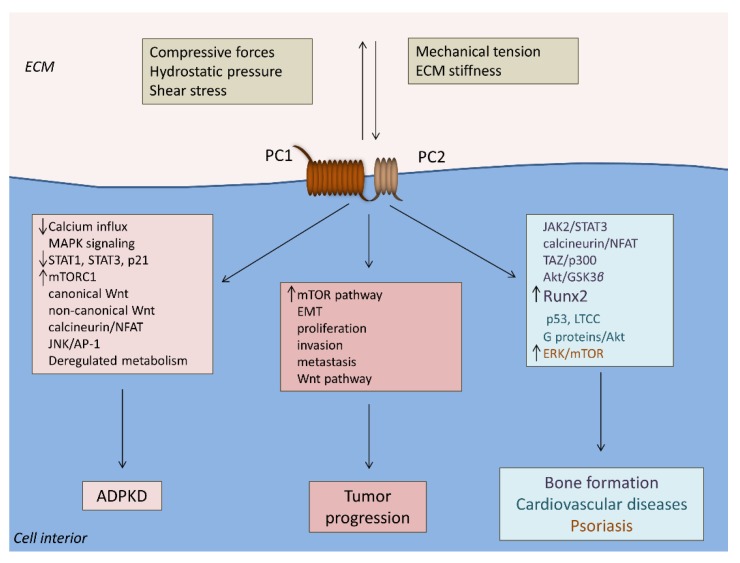
Polycystin-associated pathogenic mechanisms in human diseases. PC1 and PC2 form complexes at the cell surface and sense alterations of extracellular cell-specific mechanical forces. PC1 and PC2 loss of function induces distorted mechanotransduction that promotes ADPKD. PC1 and PC2 tissue-specific function in cancer cells facilitates tumor progression. PC1 and PC2 induce Runx2 upregulation and promote osteoblastogenesis under mechanical tension, whereas PC1 downregulation activates the mTOR pathway and mediates the properties of psoriasis in keratinocytes such as increased cell proliferation, migration, inflammation, and angiogenesis. PC1 and PC2 have also been implicated in atherosclerosis and cardiomyopathy molecular mechanisms. Abbreviations: ADPKD, autosomal polycystic kidney disease; AP-1, activator protein-1; ECM, extracellular matrix; EMT, epithelial-to-mesenchymal transition; ERK, extracellular signal-regulated kinase; GSK3β, glycogen synthase kinase 3 beta; JAK2, Janus kinase 2; JNK, c-Jun N-terminal kinase; LTCC, L-type calcium channel; MAPK, mitogen-activated protein kinase; mTOR, mammalian target of rapamycin; NFAT, nuclear factor of activated T-cells; PC1, polycystin-1; PC2, polycystin-2; Runx2, Runt-related transcription factor 2; STAT, signal transducer and activator of transcription; TAZ, transcriptional coactivator with PDZ-binding motif.

Polycystin-associated signaling pathways are implicated in ADPKD pathogenesis (Figure 2) [25]. PC1 and PC2 form complexes at the primary cilium, which represents an epithelial structure that senses mechanical cues from the fluid flow in the surface of kidney tubular cells [26]. In particular, the primary cilium bends in the presence of the urine flow and PC1 senses the alterations of the extracellular mechanical status through its large N-terminal domain. PC1 structural change stimulates PC2 and leads to an increase in the concentration of the intracellular calcium ions [25,27]. Alterations in the concentration of the intracellular calcium ions induce downstream signaling networks and changes of respective gene expressions that regulate cell proliferation and apoptosis, cell orientation, and cell differentiation [25]. In the absence of normal PC1 and PC2 expression, there is decreased flow of intracellular calcium in the cytoplasm and further disturbed signal transduction mechanisms [25]. More specifically, ADPKD cells present cystogenic calcium and subsequent cyclic adenosine monophosphate (cAMP) signaling which is mediated by the mitogen-activated protein kinase (MAPK) signaling pathway, ultimately leading to increased fluid secretion and cell proliferation. Cell growth and cell cycle are also deregulated by downregulation of STAT1, STAT3, and p21, and upregulation of mammalian target of rapamycin 1 (mTORC1) signaling. There is also deregulation of the canonical and non-canonical Wnt pathways that regulate cell proliferation and planar cell polarity, respectively [28]. PC1 also functions as an atypical GPCR. PC1 loss of function is associated with upregulation of GPCR signaling (modulation of calcineurin–nuclear factor of activated T-cells (NFAT) signaling, and c-Jun N-terminal kinase (JNK) and activator protein-1 (AP-1) activity) as was shown in *PKD1* mutant mouse proximal tubular cell cultures [29]. An ADPKD mutation (L4132Δ) in a G protein-binding region of the PC1 CTT also downregulates PC1-mediated G protein signaling, resulting in decreased basal and Gα-augmented AP-1 activity. In addition, it seems that several of the ADPKD mutations are in or around the G protein-regulating region of the PC1 CTT, so therefore the GPCR-associated function of PC1 is crucial for ADPKD pathogenesis [30]. Even though it has been proved that PC2 is required as an ion channel subunit in renal collecting duct cells, a more recent study by the same group abolishes the theory that the primary cilia mediate mechanosensation through the regulation of calcium concentration [31,32]. The researchers developed a transgenic mouse that expresses a cilia-specific calcium indicator, and they also showed that no calcium influx is triggered in primary cilia-expressing cells as a response to fluid flow [31].

Disturbed cell metabolism has lately emerged as a feature of ADPKD. The basic difference is that cyst-producing kidney cells prefer glycolysis to oxidative phosphorylation [28]. PC1 and PC2 are detected in mitochondria-associated membranes and regulate the calcium uptake by mitochondria, therefore affecting oxidative phosphorylation [16]. Polycystins also regulate energy production indirectly through the modulation of mechanisms such as AMP-activated protein kinase (AMPK), peroxisome proliferator-activated receptor alpha (PPARα), peroxisome proliferator-activated receptor gamma coactivator 1-alpha (PGC1α), calcium signaling, mTORC1, cAMP, and cystic fibrosis transmembrane conductance regulator (CFTR)-mediated ion transport [28]. In particular, in mouse embryonic fibroblasts which do not express PC1, there is increased glucose uptake and glycolysis, a process that is dependent on mTORC1 activation and suppression of AMPK activity [28]. AMPK also phosphorylates CFTR and, therefore, reduces its capacity for ion transport and promotes secretion of fluid in the cysts. In cyst-producing cells, impaired lipid metabolism and fatty acid oxidation occurs via downregulation of PPARα and its target genes, whereas other metabolic-associated features include suppression of calcium signaling, elevation of cAMP levels, and decreased expression of the mitochondrial metabolism regulator PGC1α [28]. *PKD1*^−/−^ renal epithelial cells have deregulated fatty acid metabolism, and aberrant mitochondrial morphology and function, whereas mitochondria in kidneys of ADPKD patients have morphological alterations. More specifically, mitochondria in cells lacking *PKD1* are less elongated and appear to have increased fragmentation of the mitochondrial network. A CTT cleavage product of PC1 translocates to the mitochondria matrix, and CTT expression in *PKD1*^−/−^ cells restores proper mitochondrial integrity [17].

## 4. Polycystins Participate in the Acquisition of Oncogenic Features in Cancer Cells

Cancer cells demonstrate distorted mechanisms of mechanotransduction [4]. In the primary tumor, cancer cells are subjected to increased compressive stress from alterations in the tumor’s microenvironment [33]. They are also subjected to increased mechanical tension due to the elevated rates of cell proliferation [33]. Alterations in the stiffness of the ECM rearrange the topology and orientation of cancer cells [33]. Therefore, a vicious cycle is being formed where the stiffer ECM causes alterations in mechanotransduction and gene transcription that furthers augments the rigidity of the ECM [33]. Increased rigidity of the ECM leads to integrin activation and also activation of additional proteins of the focal adhesion complexes, such as focal adhesion kinase (FAK) [4]. This leads to activation of pathways that increase the cytoskeletal tension and also the activation of proliferating pathways, such as the MAPK pathway [4]. These activations induce oncogenic gene transcription and favor the invasion and metastasis of cancer cells [4]. In particular, cancer-associated fibroblasts and other stromal cells, which are abundant in the tumor, facilitate the breach of the basal membrane and the migration of cancer cells into the circulatory system, where the circulating tumor cells (CTCs) are further subjected to vasculatory shear stress [33]. CTCs are trapped in small capillaries and extravasate into distant tissues [33].

A recent study depicts that mechanotransduction induces phenotypic alterations in prostate cancer cells and reduced sensitivity to paclitaxel [34]. Prostate cancer PC3 cells, which were cultured under soft culture conditions, presented increased mRNA expression of *E-cadherin*, decreased expression of *N-cadherin*, and increased expression of further epithelial markers. They also exhibited increases of mesenchymal markers, therefore suggesting phenotypic alterations that are associated with the transition between the mesenchymal and epithelial states [34]. There are also data presenting that melanoma cells resistant to BRAF inhibitors showed increased mechano-induced activity of the yes-associated protein 1 (YAP)/transcriptional coactivator with PDZ-binding motif (TAZ) transcription factors, and that the survival of the resistant cells was reduced after YAP/TAZ downregulation [35]. It seems that the investigation of the mechanotransducing mechanisms in oncogenesis may also have therapeutic potential according to two possible axes of application [36,37]. The first aspect involves specific protein molecules of the mechanotransducing complexes (e.g., integrins, Src, FAK) that can be targeted and tested in combination regimens. Integrins α5*β*1, α5*β*3, and α5*β*5, FAK, Src, and YAP are already being tested in clinical trials [33,36,37]. The second aspect describes new strategies that employ the biophysical properties of newly developed mechano-induced vector systems in order to selectively target metastases. This can be achieved with mechano-induced vectors of mesenchymal stem cells, which take advantage of the composition of the ECM and the generation of corresponding mechanical cues in order to selectively deliver the chemical compounds [36,37,38].

In this context, polycystins emerge as novel prognostic and diagnostic tools in cancer (Table 1) [5]. We already know that ADPKD shares common features with oncogenesis, and also the first nationwide cohort study in Taiwan showed that patients with polycystic kidney disease without end-stage renal disease have increased risks of developing liver, colon, and kidney cancers [39,40]. PC1 and PC2 may present different ways of functioning in cancer cells and, therefore, different ways of contributing to cancer development. PC1 is expressed in colorectal cancer (CRC) cells and can potentially mediate cell-to-cell and cell-to-ECM interactions either alone or in a complex with PC2. PC1 can thereby facilitate epithelial-to-mesenchymal transition (EMT), invasion, and metastasis of cancer cells. We already described that PC1 also generates transcriptionally active CTT fragments [13]. Therefore, PC1 could affect gene transcription through its CTT that translocates to the nucleus. This could happen through the interactions of PC1 with mechano-induced transcription factors, such as YAP and TAZ [5,37]. Nevertheless, it has already been found that PC1 interacts with TAZ in osteoblasts [41].

Specifically in CRC SW480 cells, PC2 overexpression is accompanied by the activation of the mTOR pathway [42]. On the other hand, PC1 overexpression in invasive HCT116 cells is followed by a gene expression profile that promotes EMT, and therefore invasion and metastasis [42]. In corroboration, in a CRC HT29 xenograft model, treatment with an inhibitor of the PC1 N-terminal end caused tumor necrosis, and decreased cell proliferation and EMT, as well as enhancement of apoptosis [42]. In human CRC tumors, elevated PC1 expression was associated with mucinous carcinomas of poor prognosis, increased depth of invasion, and was also highlighted as an independent factor without relapse [42]. Based on these findings, it seems that polycystins may be on the crossroad between extracellular mechanical cues (alterations in matrix rigidity and matrix proteins) and intracellular oncogenic mechanical signaling and gene transcription (Figure 2) [43]. Since epigenetic regulation is crucial for the molecular classification and prognosis in CRC, it would be interesting to investigate the methylation status of the PC1-encoding gene *PKD1*, a gene that is frequently hypermethylated, in CRC samples as an additional diagnostic tool [44]. *PKD1* has also been found to be among twenty high-risk mutant genes with RNA sequencing in human prostate cancer samples [45]. An older study highlights that PC1 overexpression facilitates adherence and aggregation in SW480 CRC, A549 lung, and HepG2 liver cancer cells. This overexpression inhibits cancer cell migration and invasion, possibly through the Wnt pathway [46]. A second study by the same group revealed that PC1 overexpression induces caspase-dependent increased apoptosis and cell cycle arrest at the G0/G1 phases without any notable impact on cell proliferation [47]. *PKD2* silencing in B16 mouse melanoma cells also abrogates cell-to-cell but not cell-to-ECM adhesion [48]. Collectively, these findings suggest that polycystins probably have tissue-specific functions in cancer cells and, furthermore, that polycystins may behave differently between the initial proliferating tumor and the invading cancer cells that feature aggressive phenotypes (Figure 2). PC1 and PC2 are also implicated in other types of tumor cells, such as osteosarcoma cells that feature an intense mechanotransducing network [49].

## 5. Polycystins in Bone Loss, Cardiomyopathies, and Other Pathophysiologies

Mechanotransduction regulates the differentiation of osteoblasts, as well as adipogenesis and bone mass formation. This role is facilitated through the polycystin proteins and mechanosensitive structures such as the primary cilia, integrins, and downstream signaling pathways associated with PC1 and PC2 (Table 1) (Figure 2) [50,51]. Stable *PKD1* knockdown in the mouse osteoblastic cell line MC3T3-E1 is associated with decreased expression of osteoblastic differentiation markers, among them runt-related transcription factor 2 (Runx2) [52]. It is also known that PC1 activates Janus kinase 2 (JAK2) through the PC1-CTT under mechanical loading in primary osteoblast-like cells. This activation leads to STAT3 translocation to the nucleus, which upregulates the bone-specific *runx2* gene expression [53]. In corroboration, it has been documented that PC1 also induces Runx2 expression under mechanical strain via the calcineurin/NFAT pathway [54]. Application of mechanical stretch and increased stiffness of the ECM stimulates the formation of a nuclear complex between the mechano-induced co-activator TAZ, PC1, and PC2, which is associated with increased osteoblastogenesis via activation of *runx2* expression and repressed adipogenesis through deactivation of PPARγ [41]. In addition, the PC1-CTT physically interacts with TAZ and recruits the p300 transcriptional co-regulator in order to enhance the expression of Runx2 in pre-osteoblasts [55]. The application of hydrostatic pressure itself in chondrogenic ATDC5 cells regulates the expression of Runx2 and leads to increased mRNA levels of *PKD1* and *PKD2*, thus implying a potential positive feedback between polycystins and *runx2* gene expression [56].

On the other hand, PC2 is also a mechanosensor of extracellular mechanical cues in osteoblasts. PC2 induces increases of intracellular Ca^2+^, Akt phophosrylation/activation, and subsequent phosphorylation of glycogen synthase kinase 3 beta (GSK3*β*). GSK3*β* phosphorylation abrogates the deactivation of *β*-catenin and induces *β*-catenin translocation to the nucleus in order to promote *runx2* gene transcription [57]. *PKD2* inactivation in mature mouse osteoblasts has been found to lead to osteopenia. *PKD2*-deficient mice also exhibit reduced mRNA expression of several osteoblast-specific genes and diminished expression in adipocyte-specific markers [58].

PC1 and PC2 are expressed in a variety of tissues of the cardiovascular system, and they have been implicated in the pathophysiology of extrarenal vascular manifestations of ADPKD, as well as other cardiovascular diseases (Table 1) [59]. Primary cilia in the endothelium sense fluid flow, and PC1 regulates calcium influx along with the production of the vasodilator nitric oxide [60]. Low shear stress in endothelial cells increases the expression of PC1 and PC2, which is accompanied by activation of p53. PC1 expression is also higher in the aortic plaques of atherosclerotic patients and is associated with dismal prognostic factors such as dyslipidemia, diabetes mellitus, hypertension, and carotid stenosis (Figure 2) [61]. In vascular smooth muscle cells, decreased expression of PC1 or increased expression of PC2 suppresses the activity of stretch-activated ion channels. Based on this finding, it seems that the altered expression ratio of PC1/PC2 in favor of PC2 regulates pressure sensing in blood vessels [62].

Apart from the endothelium, PC1 was also proved to serve as a mechanosensor in cardiomyocytes. PC1 and PC2 are expressed in cardiomyocytes in 5-week human embryos [63]. The L-type calcium channel (LTCC) is an important regulator of cardiac contractility. Neonatal rat ventricular myocytes subjected to mechanical stretch need intact expression and function of PC1 to trigger hypertrophy of the cells in an LTCC-dependent manner and with respective adequate α1C protein expression [64]. Furthermore, in order to clarify the molecular mechanism by which PC1 regulates LTCC protein stabilization, the same group subjected the same cells to mechanical stretch and proved that LTCC stabilization depends on the PC1-dependent activation of Akt kinase (Figure 2). In particular, Akt activation further depends on the G protein receptor activity of PC1 and the associated Gβγ subunit of a heterotrimeric Gi/o protein [65]. PC2 is also implicated in cardiovascular anomalies. *PKD2* mutant zebrafish exhibit low cardiac output, and the *PKD2* mutant hearts show aberrant intracellular calcium cycles. In the same study, dilated cardiomyopathy was detected in ADPKD patients [66]. Furthermore, PC2 deficiency has a cumulative impact, since it causes alterations in calcium-associated proteins that become prolonged and are enhanced with aging, as was shown in *PKD2*^+/−^ mice. Older mice also had signs of dilated cardiomyopathy and decreased left ventricular ejection fractions [67].

## 6. Conclusions

The role of mechanical stresses and alterations of mechanical interactions at the molecular level have formed a distinct field in the investigation of human and microorganism development. Lately, this subject of mechanobiology has tended to also form a distinct field of research for the development and progression of human diseases. Mechanotransduction is a core process during these functions, which is subjected to various alterations in human pathology, as has been recently documented. Thorough investigation of the mechanotransducing networks and the respective molecular mechanisms can potentially provide new diagnostic tools, as well as novel therapeutic targets. The main aspects of this potential include the emergence of new targets but also the employment of the mechanical features of cells in favor of selective drug delivery.

Although polycystins have been extensively explored in ADPKD, it seems that their biological behavior has reference to other human pathophysiologies (Table 1). For example, psoriasis is an inflammatory skin disease where the application of mechanical stress triggers the onset of the psoriatic plaque formation [68]. PC1 was recently found to be downregulated in psoriatic tissues, and this downregulation promotes psoriatic features through an extracellular signal-regulated kinase (ERK)-dependent mTOR pathway activation (Figure 2) [69]. Experimental data reveal that polycystins may not be engaged to molecular mechanisms of human pathology in the same way as they are in ADPKD, which is mostly by loss of their function. Therefore, it seems that polycystins have cell-specific biological behavior. They may also have different contributions according to the progression of the disease, for example different impacts during the onset and during the invasion of the tumor. Overall, the “threshold” model of polycystins in ADPKD, meaning that they are pathogenic when their function falls below or exceeds a specific limit, probably describes their contribution to other diseases. Conclusively, polycystins comprise a promising family of proteins for further investigation regarding disturbed mechanisms of mechanotransduction in human diseases.

## Figures and Tables

**Table 1:** The impact of polycystin deregulation in certain pathophysiologies.

Pathophysiology	Pathogenic Mechanism
Autosomal dominant polycystic kidney disease	*PKD1* and *PKD2* loss-of-function mutations Aberrant sensing of the urine flow Deregulated calcium influx Pathogenic signaling promotes cystogenesis
	Deregulated cell metabolism
Cancer	PC2 overexpression activates the mTOR pathway in CRC
	PC1 overexpression promotes EMT, invasion and metastasis in CRC
	*PKD1* among high-risk mutant genes in prostate cancer
Bone formation	*PKD1* and *PKD2* downregulation inhibits osteoblastogenesis
	PC1 under mechanical loading leads to increased osteoblastogenesis through various mechanisms
Cardiovascular diseases	PC1 overexpression in endothelial cells follows disturbed shear stress and is associated with atherosclerosis
	*PKD1* and *PKD2* downregulation is associated with cardiomyopathy
Psoriasis	Loss of PC1 expression promotes psoriasis via ERK and mTOR activation

Abbreviations: CRC, colorectal cancer; EMT, epithelial-to-mesenchymal transition; ERK, extracellular signal-regulated kinase; mTOR, mammalian target of rapamycin; PC1, polycystin-1; PC2, polycystin-2; PKD1, polycystic kidney disease 1; PKD2, polycystic kidney disease 2.

## Abbreviations

ADPKD	Autosomal polycystic kidney disease
AMPK	AMP-activated protein kinase
AP-1	Activator protein-1
cAMP	Cyclic adenosine monophosphate
CFTR	Cystic fibrosis transmembrane conductance regulator
CHOP	C/EBP homologous protein
CRC	Colorectal cancer
CTCs	Circulating tumor cells
CTT	C-terminal tail
ECM	Extracellular matrix
EMT	Epithelial-to-mesenchymal transition
ERK	Extracellular signal-regulated kinase
FAK	Focal adhesion kinase
GPCR	G-protein coupled receptor
GSK3β	Glycogen synthase kinase 3 beta
JAK2	Janus kinase 2
JNK	c-Jun N-terminal kinase
LTCC	L-type calcium channel
MAPK	Mitogen-activated protein kinase
mTOR	Mammalian target of rapamycin
NFAT	Nuclear factor of activated T-cells
PC1	Polycystin-1
PC2	Polycystin-2
PGC1α	peroxisome proliferator-activated receptor gamma coactivator 1-alpha
PHD3	Prolyl hydroxylase domain containing protein EGLN3
PKD1	Polycystic Kidney Disease 1
PKD2	Polycystic Kidney Disease 2
PPARα	Peroxisome proliferator-activated receptor alpha
Runx2	Runt-related transcription factor 2
STAT	Signal transducer and activator of transcription
TAZ	Transcriptional coactivator with PDZ-binding motif
TCF	T-cell factor
YAP	Yes-associated protein 1
